# Moving CNS axon growth and regeneration research into human model systems

**DOI:** 10.3389/fnins.2023.1198041

**Published:** 2023-06-22

**Authors:** Bo P. Lear, Darcie L. Moore

**Affiliations:** Department of Neuroscience, University of Wisconsin-Madison, Madison, WI, United States

**Keywords:** axon growth, axon regeneration, direct reprogramming, hPSCs, reprogramming, development

## Abstract

Axon regeneration is limited in the adult mammalian central nervous system (CNS) due to both intrinsic and extrinsic factors. Rodent studies have shown that developmental age can drive differences in intrinsic axon growth ability, such that embryonic rodent CNS neurons extend long axons while postnatal and adult CNS neurons do not. In recent decades, scientists have identified several intrinsic developmental regulators in rodents that modulate growth. However, whether this developmentally programmed decline in CNS axon growth is conserved in humans is not yet known. Until recently, there have been limited human neuronal model systems, and even fewer age-specific human models. Human *in vitro* models range from pluripotent stem cell-derived neurons to directly reprogrammed (transdifferentiated) neurons derived from human somatic cells. In this review, we discuss the advantages and disadvantages of each system, and how studying axon growth in human neurons can provide species-specific knowledge in the field of CNS axon regeneration with the goal of bridging basic science studies to clinical trials. Additionally, with the increased availability and quality of ‘omics datasets of human cortical tissue across development and lifespan, scientists can mine these datasets for developmentally regulated pathways and genes. As there has been little research performed in human neurons to study modulators of axon growth, here we provide a summary of approaches to begin to shift the field of CNS axon growth and regeneration into human model systems to uncover novel drivers of axon growth.

## 1. Introduction

Damage to adult central nervous system (CNS) neuronal projections called axons, in spinal cord injury (SCI) or optic nerve injury for example, results in little to no regeneration following damage. SCI is a devastating injury that results in life-long disability with high rates of morbidity and mortality, resulting in reduced quality of life, and a lifetime economic burden of approximately 2–4 billion dollars (Bennett and Emmady, 2023). The lack of regeneration in adult CNS axons is due to both an inhibitory environment and a lack of intrinsic axon growth in the neurons themselves (Curcio and Bradke, 2018; Zheng and Tuszynski, 2023). Interestingly, there is an age-dependent decrease in axon growth and regeneration ability in rodent CNS neurons *in vitro* and *in vivo* around the time of birth (Bregman et al., 1989; Chen et al., 1995; Dusart et al., 1997; Goldberg et al., 2002; Gwak et al., 2004; Blackmore and Letourneau, 2006; Genovese et al., 2006; Siegenthaler et al., 2008; Moore et al., 2009; Mar et al., 2014). This largely has been considered to be due to a downregulation of pro-regenerative networks and an upregulation of inhibitory gene expression networks in addition to developmental changes in the environmental niche (Gao et al., 2004; Arlotta et al., 2005; Wang et al., 2007; Moore et al., 2009; Geoffroy et al., 2016; Lu et al., 2020). Despite immense knowledge gained through identifying modulators of axon regeneration in rodents, there is currently no FDA-approved treatment for SCI (Perrin, 2014; Dietz et al., 2022). The lack of concordance of results between preclinical rodent studies and human clinical trials could be the result of the divergence of not only the human and mouse transcriptome, but also *cis*-regulatory regions (e.g., promoters, enhancers; Yue et al., 2014). Thus, it is important to establish human studies to determine the conservation across species of critical regulators of axon growth. Until recently, our ability to ask these questions was limited by the inability to acquire and culture human neurons of all ages. In this review, we summarize our current knowledge of developmentally-regulated axon growth pathways in the rodent CNS, human *in vitro* and *in vivo* models to study axon regeneration, and finally human cortical tissue transcriptomic and proteomic datasets available for mining developmentally-regulated genes in human CNS neurons.

## 2. An age-dependent decline in rodent CNS axon regeneration

It has been well-established that developmental age alters the response of rodent CNS neurons to extrinsic and intrinsic factors that limit adult but not embryonic axon growth and regeneration (Chen et al., 1995; Bandtlow and Loschinger, 1997; Dusart et al., 1997; Fawcett, 1997; Goldberg et al., 2002; Blackmore and Letourneau, 2006; Blackmore et al., 2012; Venkatesh et al., 2016; Curcio and Bradke, 2018; Venkatesh et al., 2018; Wang Z. et al., 2018; Zheng and Tuszynski, 2023). Extrinsically, CNS myelin contains inhibitory proteins, such as NOGO, myelin-associated glycoprotein (MAG), and oligodendrocyte myelin glycoprotein (OMGP) that limit adult axon CNS regeneration through collapsing growth cones following injury, but not embryonic CNS neurons (Waxman and Foster, 1980; Foran and Peterson, 1992; Filbin, 2003; Yiu and He, 2006). Further, with aging, there is an attenuated ability for local microglia and macrophages to remove myelin debris resulting in prolonged inflammation (Safaiyan et al., 2016; Cantuti-Castelvetri et al., 2018). After the inflammation has stabilized, a glial scar forms, which is both a mechanical barrier to axon regeneration, and also contains proteins inhibitory to axon growth such as chondroitin sulfate proteoglycans (CSPGs) released by reactive astrocytes and other cell types locally (Snow et al., 1990; McKeon et al., 1999; Becker and Becker, 2002; Jones et al., 2003; Tang et al., 2003; Yiu and He, 2006; Li et al., 2020). Further, in rodent SCI models there is increased inflammation in older animals as represented by an increase in monocyte-derived macrophages at the lesion site (Stewart et al., 2021).

Intrinsically, development and age influence various pathways in the neurons, from epigenome to transcriptome to translatome. Originally, developmentally-regulated transcription factors (TFs) were attractive targets because they can regulate an abundance of genes. For example, Krüppel-like factors (KLF) 6 and KLF7, which promote embryonic axon growth, are downregulated postnatally (Moore et al., 2009; Blackmore et al., 2012; Wang Z. et al., 2018; Kramer et al., 2021), whereas other members of the KLF family, such as KLF4 and 9, are upregulated developmentally, and inhibit axon growth and regeneration (Moore et al., 2009; Apara et al., 2017; Galvao et al., 2018; Trakhtenberg et al., 2018; Avila-Mendoza et al., 2020; Xu et al., 2021). Knockdown of *Klf4* and *9* can increase axon growth in adult corticospinal tract (CST) neurons and retinal ganglion cells (RGCs; Moore et al., 2009; Apara et al., 2017; Galvao et al., 2018; Trakhtenberg et al., 2018). KLF9’s inhibitory function is partially mediated through the MAPK pathway as the inhibition or inactivation of c-Jun N-terminal kinase 3 (JNK3) and Dual-specificity phosphatase 14 (DUSP14) abolish KLF9’s inhibitory role in RGC axon regeneration (Apara et al., 2017; Galvao et al., 2018). KLF4 acts through binding signal transducer and activator of transcription 3 (STAT3) to block STAT3’s DNA-binding activity, limiting expression of its downstream regeneration-associated genes (RAGs) (Qin et al., 2013). In contrast to KLF4, the pro-regenerative KLF6 has cooperative roles with STAT3 to promote regeneration through the co-occupancy of similar regulatory regions of DNA (Wang Z. et al., 2018; Kramer et al., 2021). These studies suggest that in the CNS, both developmentally regulated growth-promoting and growth-repressing TFs act to drive changes in axon growth and regenerative abilities.

In addition to developmentally regulated TFs, cell signaling proteins such as insulin-like growth factor, cytochrome P450, and cyclic adenosine monophosphate (cAMP) levels are all downregulated during development (Gao et al., 2004; Arlotta et al., 2005; Wang et al., 2007). cAMP is an example where intrinsic and extrinsic factors collide, as cAMP downregulation in cerebellar and dorsal root ganglion (DRG) neurons at postnatal day (P)3–4 leads to increased inhibition of axon growth by myelin (Cai et al., 2002; Gao et al., 2004), due to the reduced phosphorylation of cAMP- response element-binding protein, and thus its effect on downstream genes such as arginase I and interleukin 6 (Cai et al., 2002; Redmond et al., 2002; Gao et al., 2004; Cao et al., 2006; Deng et al., 2009). In these examples, modulation of these age-dependent factors has been shown to improve CNS axon growth and regeneration in rodent models.

The mammalian target of rapamycin (mTOR) pathway is a central pathway that promotes growth through translational regulation (Ma and Blenis, 2009; Saxton and Sabatini, 2017). During CNS neuronal development, there is a downregulation of mTOR pathway activity, detected through a decrease in phospho-S6 signal, a marker of mTOR pathway activation (Liu et al., 2010; Teotia et al., 2019). The deletion of negative regulators of the mTOR pathway such as phosphatase and tensin homolog (*Pten*) and tuberous sclerosis 1 (*Tsc1*), promotes robust axon regeneration in RGCs and CST neurons (Park et al., 2008; Liu et al., 2010). However, later studies found that whereas *Pten* deletion in young animals (6 weeks) resulted in a strong regenerative phenotype, the same deletion in middle-aged animals (12–18 months) resulted in limited CST neuron axon regeneration (Geoffroy et al., 2016, 2017). This is thought to be due to the upregulation of eukaryotic translation initiation factor 4E-binding protein 1 (4E-BP) during aging which promotes cap-dependent translation of downstream mRNAs. mTORC1 is a repressor of 4E-BP, thus *Pten* deletion should lead to increased activation of mTORC1, and increased repression of 4E-BP. However, because 4E-BP expression is upregulated with age, this prevents the downstream effects of *Pten* deletion, leading to the lack of regeneration in older animals (Yang et al., 2014; Geoffroy et al., 2016). Thus, *Pten* deletion, one of the hallmark gene manipulations that promotes robust axon regeneration either singly (Park et al., 2008, 2010; Liu et al., 2010; Du et al., 2015; Geoffroy et al., 2016) or in combination with other treatments (Kurimoto et al., 2010; Sun et al., 2011; de Lima et al., 2012; Lewandowski and Steward, 2014; O’Donovan et al., 2014; Ohtake et al., 2014; Geoffroy et al., 2015; Jin et al., 2015; Lim J. H. et al., 2016; Weng et al., 2018; Huang et al., 2019; Xie et al., 2022), is hindered in the aged rodent CNS due to age-dependent changes. Other mTOR pathway regulators are developmentally regulated such as LIN28A, an RNA-binding protein, which is downregulated by early postnatal development. Its upregulation in both CST neurons and RGCs results in robust axon regeneration and improved motor function in the injured mice (Nathan et al., 2020).

It has been hypothesized that the reduced effectiveness of known pro-regenerative transcription factors in aging rodent models may be due to changes in chromatin accessibility. For example, overexpression of pro-regenerative transcription factors such as JUN and STAT3 have attenuated effects on adult CST axon regeneration, despite their regenerative phenotypes when overexpressed in early postnatal neurons *in vitro* (Lerch et al., 2014; Mehta et al., 2016; Venkatesh et al., 2018). Similarly, with chromatin accessibility analysis, the pro-regenerative TF KLF7 had reduced DNA binding in adult cortical neurons due to changes in chromatin structure, and thus decreased DNA accessibility (Blackmore et al., 2012; Venkatesh et al., 2018; Pita-Thomas et al., 2021). These examples of reduced effectiveness of TFs in adult neurons may be due to a general developmental decrease in promoter accessibility of RAGs (Gaub et al., 2011; Venkatesh et al., 2016). A possible mechanism underlying these changes in DNA accessibility is through an age-dependent decline in histone acetyltransferase CBP/p300 activity, which relaxes chromatin and allows for transcriptional initiation (Gaub et al., 2010, 2011). Overexpression of p300 in adult injured RGCs, and to a lesser extent in CNS upper motor neurons, promotes axon regeneration through increased p300 interaction and acetylation of the promoter regions of RAGs, leading to increased DNA accessibility and transcription (Gaub et al., 2010, 2011; Muller et al., 2022). On a global scale, transient pulsing of 3 out of the 4 Yamanaka reprogramming factors (octamer-binding transcription factor 4 (OCT4), sex-determining region Y box 2 (SOX2), and KLF4) in adult RGCs reverted these neurons to an embryonic state, rejuvenating the chromatin landscape, and resulting in increased optic nerve regeneration post-injury (Lu et al., 2020). This increased regeneration following transient reprogramming was mediated by tet methylcytosine dioxygenase 1 (TET1) and TET2-dependent DNA demethylation shifting the epigenetic landscape from an adult to an embryonic state. Thus, a developmental switch in DNA accessibility may act to limit CNS axon regenerative ability in the adult.

Tremendous progress has been made in identifying intrinsic and extrinsic rodent regulators of CNS axon regeneration, yet we do not know if these are conserved in human CNS neurons. For example, do pathways like mTOR and cAMP mediate similar effects on human axon growth? Is CNS axon growth and regeneration ability also developmentally-regulated in humans, and if so, what are the underlying changes that drive this phenotype? These are open questions where little has been studied in the context of human model systems.

## 3. Models to study human axon growth

In the last two decades, with the advent of human pluripotent stem cell (hPSC) technology which includes both human embryonic stem cells (hESCs) and induced pluripotent stem cells (iPSCs), and direct reprogramming technologies, we have seen an increase in human disease and therapeutic models that can now be applied to studies of axon growth and regeneration.

### 3.1. Human pluripotent stem cell (hESC and iPSC) models

**Figure 1:**
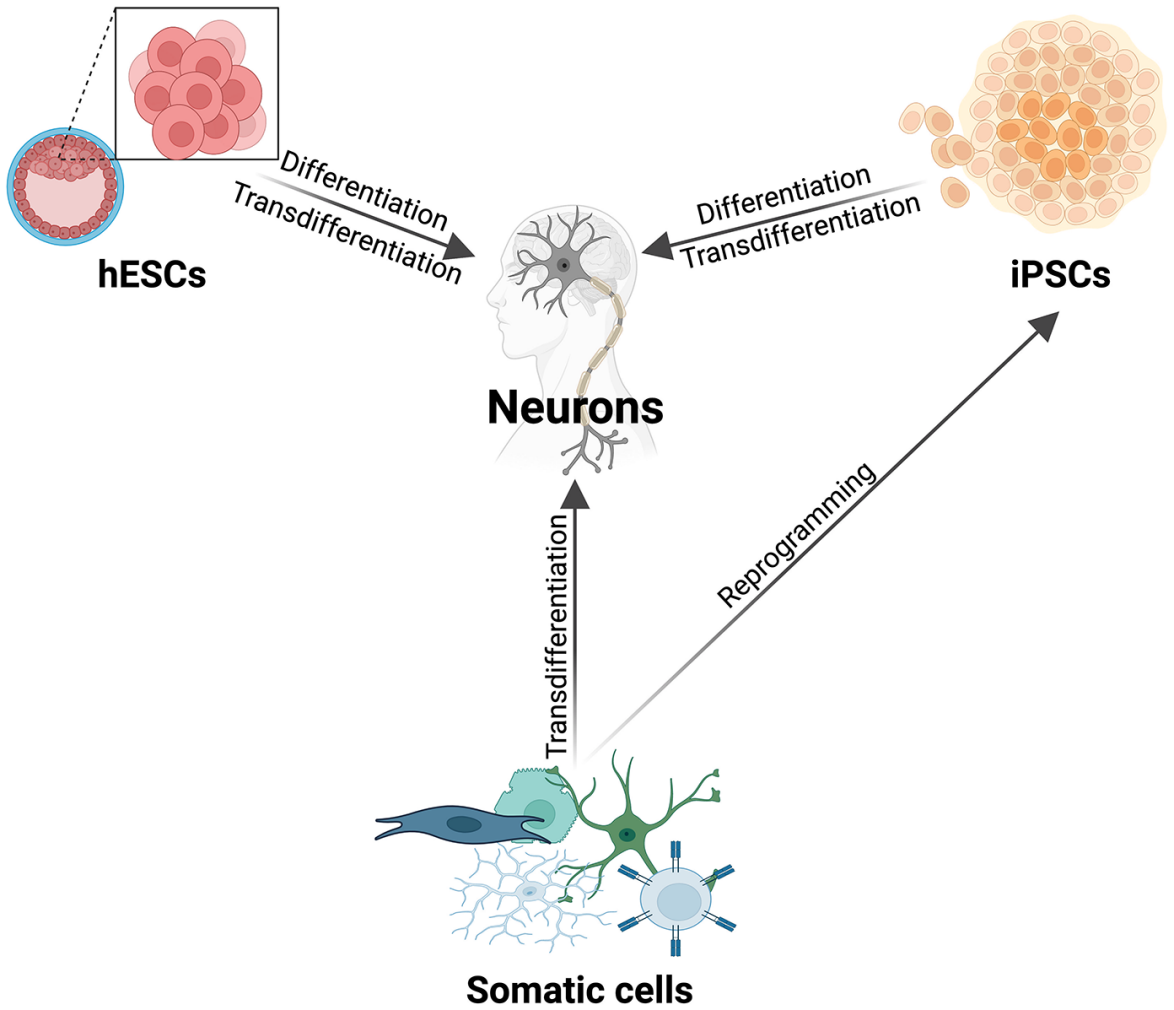
The most commonly used methods to make human neurons. Schematic illustrating the major methods of differentiating and direct reprogramming of various cell types to human neurons (hESCs, iPSCs, somatic cells). In addition to the listed cell types for transdifferentiation, there are a multitude of donor cells that can be directly reprogrammed to human neurons [reviewed in Kim et al. (2021)].

HESCs are embryonic stem cells isolated from the inner cell mass of a human blastocyst which can be cultured *in vitro*, and differentiated into a wide variety of cells, including neurons (Figure 1; Shamblott et al., 1998; Thomson et al., 1998; Keller and Snodgrass, 1999). To avoid the ethical concerns of the source of hESCs, iPSCs have become a strong alternative for creating human neurons (Figure 1). IPSCs are generated from somatic cells using the overexpression of specific proteins resulting in cellular, epigenetic, and transcriptomic rejuvenation, and returning the original adult cell to a prenatal epigenetic and cellular age.

HPSCs can be differentiated into diverse neural precursors, neurons, and glial subtypes following the addition of various combinations of small molecules and growth factors that mirror developmental patterning [reviewed in (Kramer et al., 2013; Chuang et al., 2015; Tao and Zhang, 2016)]. Both hPSCs and hPSC-derived neural progenitor cells (NPCs) are highly proliferative, allowing for the constant expansion of the starting population, and the generation of large numbers of neurons. However, depending on the desired final cell type, it can take weeks to months for neurons to fully differentiate and mature because this differentiation paradigm mimics development. A shorter process to generate these subtypes of neurons is the forced overexpression (direct reprogramming) of neurogenic transcription factors or microRNAs in hPSCs without the natural progression of development (Mertens et al., 2015, 2018; Tang et al., 2017).

HPSC-derived cells can be cultured in 2D or 3D, with 2D systems allowing for easy analysis of individual neuronal morphology, whereas 3D models have the advantage of mirroring the *in vivo* environment (Seo et al., 2022). A small number of studies have used hPSC-derived neurons in 2D culture for high content screens to test for compounds that could modulate intrinsic axon growth, as well as for the impact of various substrates and scaffolds on axon growth [hESC-derived neurons: (Lam et al., 2010; Shin et al., 2010), iPSC-derived neurons: (Sirenko et al., 2014; Hancock et al., 2015; Sherman and Bang, 2018)]. Recently, researchers have generated 3D motor column organoids to model amyotrophic lateral sclerosis (ALS). These 3D organoids contain a mixture of motor neurons and interneurons, with key elements of spinal cord organization replicated in this model such as motor neuron axons bundles exiting the organoid together, similar to a motor nerve fascicle, and ventral interneuron localization (Seo et al., 2022). Further, assembloids, generated from organoids and spheroids of different structures such as cerebral cortex, hindbrain/spinal cord, and skeletal muscle can be assembled together, in efforts to model the entire cortex to muscle functional circuitry (Giandomenico et al., 2019; Andersen et al., 2020).

While all hPSC-derived models do a good job of modeling developmental ages due to the rejuvenated age of hPSC-differentiated progeny, this may be a disadvantage if one wants to study adult human neuron axon growth and regeneration.

### 3.2. Human cells as treatment: *in vivo* use of hESC- and iPSC-derived neurons

The most common application of hPSC-differentiated neurons is therapeutic transplantation for neurodegenerative diseases. In Parkinson’s disease (PD), for example, there has been great progress with midbrain dopaminergic progenitor transplantation to replace the endogenous degenerated neurons in animal models (Wang Y. K. et al., 2018; Piao et al., 2021; Tao et al., 2021; Xiong et al., 2021), and now in clinical trials (Kim et al., 2022). These hPSC-derived midbrain dopaminergic progenitors transplanted into the midbrain of a PD mouse not only extend their axons to different targets in the brain, but are also capable of integrating into the local neuronal circuitry, resulting in moderate recovery of PD-induced motor deficits (Xiong et al., 2021). Similar results were seen with autologous transplantation of iPSCs in non-human primates (Tao et al., 2021).

Transplantation has also been used in efforts to treat SCIs. Early studies transplanted rodent embryonic cortical neurons with high intrinsic growth potential into the adult rodent cortex, and observed neuron grafts extending long-range projections and forming synapses with cortical and subcortical structures (Fricker-Gates et al., 2002; Gaillard et al., 2007; Gaillard and Sauve, 2007; Falkner et al., 2016). With the advent of hPSC technology, scientists have now generated chimeric animals through transplantation of human cells in rodent and non-human primate spinal cords (Nori et al., 2011; Lu et al., 2012, 2014a,b; Kadoya et al., 2016; Li and Chen, 2016; Nagoshi and Okano, 2017; Dulin et al., 2018; Okubo et al., 2018; Rosenzweig et al., 2018; Kumamaru et al., 2018a,b; Kitagawa et al., 2022). For example, Wertheim and colleagues generated hPSC-derived motor neurons and created hydrogels from porcine extracellular matrix (ECM) for transplantation into rodent spinal cord at the site of a SCI. This transplant resulted in enhanced cell survival, reduced inflammation and gliosis at the lesion site, and overall improved motor outcomes (Wertheim et al., 2022). Transplantation of hPSC-derived progenitors and neurons to the injury site not only alters the local environment to be growth-permissive, but also produces neurons that can integrate into the host neural circuitry, acting as a relay to improve recovery post-SCI (Cizkova et al., 2007; Usvald et al., 2010; van Gorp et al., 2013; Lu et al., 2014b, 2017; Okubo et al., 2018; Rosenzweig et al., 2018; Poplawski et al., 2020). These are exciting prospects, however, additional longitudinal studies need to be performed to evaluate how the transplantation of hPSC-derived NPCs alter the local environment and circuitry over time. HPSC-derived NPCs recently have moved into ongoing clinical trials for treatment of patients with SCIs through transplantation at the site of injury (Sugai et al., 2021; Kim et al., 2022), yet a key limitation in moving from animal models to human models is the need to generate clinical grade hPSCs. This process is extremely laborious and expensive, especially when generating autologous hPSCs from patient cells (Kim et al., 2022). As a result, scientists have been developing HLA-compatible iPSC lines to cover most of the world’s population to reduce the need to generate autologous hPSCs to prevent immune rejection (Rim et al., 2018; Jang et al., 2019; Yoshida et al., 2023). The potential for large-scale production of hPSC-derived neurons in combination with their high intrinsic axon growth potential and plasticity supports these cells as a good model for therapeutic treatments.

### 3.3. Direct reprogramming to create age-specific human neurons

To ask if developmental and age-specific regulators drive changes in human CNS axon regeneration requires the use of a human neuronal model system that maintains age. Direct reprogramming is the conversion of one somatic cell type into another, or transdifferentiation (Figure 1; Mertens et al., 2015; Tang et al., 2017; Xu et al., 2020; Vasan et al., 2021). This process preserves both the epigenetic and transcriptomic age signature, and cellular aging components of the donor cell (Mertens et al., 2015; Tang et al., 2017). This is dissimilar to differentiated cells from hPSCs, as both hESCs and iPSCs have epigenetic signatures consistent with a prenatal age (Takahashi et al., 2007; Yu et al., 2007; Horvath, 2013; Kramer et al., 2013; Mertens et al., 2015, 2021; Chen et al., 2019; Piao et al., 2021).

In recent years, *in vitro* directly reprogrammed neurons have become a powerful tool to study the molecular underpinnings of aging in neurons, and to model neurodegenerative diseases (Liu et al., 2014; Fiesel et al., 2015; Lim S. M. et al., 2016; Liu M. L. et al., 2016; Victor et al., 2018; Mertens et al., 2021; Vasan et al., 2021; Oh et al., 2022). Direct reprogramming can be achieved through overexpression of key transcription factors (El Wazan et al., 2019), overexpression of microRNAs (Yoo et al., 2011; Victor et al., 2014; Huh et al., 2016; Abernathy et al., 2017; Cates et al., 2021), genome editing with CRISPR/Cas9 (Chakraborty et al., 2014; Chavez et al., 2015; Savell et al., 2019), and small molecules (Cheng et al., 2015; Dai et al., 2015; Li et al., 2015; Zhang et al., 2015; Gao et al., 2017; Wan et al., 2018; Yang et al., 2019). Additionally, *in vitro* direct reprogramming can transdifferentiate various cell types, such as fibroblasts, astrocytes, and even T-cells (Zhang et al., 2015; Gao et al., 2017; Tanabe et al., 2018; Wang et al., 2021) into diverse neuronal types, such as glutamatergic (Ambasudhan et al., 2011; Pang et al., 2011; Yoo et al., 2011; Aydin et al., 2019), dopaminergic (Caiazzo et al., 2011; Pfisterer et al., 2011), spinal lower motor (Son et al., 2011; Liu L. et al., 2016; Tang et al., 2017), cholinergic (Liu et al., 2013), serotonergic neurons (Vadodaria et al., 2016; Xu et al., 2016), and RGCs (Wang et al., 2020). One of the strengths of this system is that the age of the original cell is retained, making it a better model to study age-specific effects. For example, Huntington’s disease (HD) patient fibroblast-induced neurons showed mutant huntingtin (HTT) aggregates and associated mitochondrial and DNA defects, but patient fibroblasts reprogrammed first to iPSCs, then transdifferentiated into neurons, did not show any abnormalities (Victor et al., 2018). This was also recapitulated in another HD study with autophagy (Oh et al., 2022). In the context of axon growth, this reprogramming strategy enables the study of adult human neurons, however, a major limitation of this system is the low yield of neurons compared to hPSC-derived, and the limited starting material due to passage exhaustion of somatic cells.

Each model discussed above has pros and cons based on the specific scientific question being asked. HPSC-derived neurons may be the best model for SCI and optic neuropathy transplantation, or high content screens for modulators of axon growth. However, direct reprogramming is a better strategy for studying age-specific regulation of axon outgrowth.

### 3.4. Human cortical tissue datasets

Large sequencing datasets from human brain are useful repositories for identifying gene expression changes in human CNS neurons during development and throughout aging. Global consortiums have been formed with the goal of identifying transcriptomic and proteomic changes across development and pathological states (PsychENCODE, BrainSpan, Brainseq, SpaceTx, Human cell atlas, etc.). Using bulk RNA sequencing (RNA-seq) and microarrays, multiple studies have published developmentally-regulated transcripts and their expression trajectories across time in various brain regions such as the dorsolateral prefrontal cortex (Breen et al., 2018; Werling et al., 2020), prefrontal cortex (Weickert et al., 2009; Colantuoni et al., 2011; Jaffe et al., 2015), and other brain regions (Johnson et al., 2009; Pletikos et al., 2014). These studies can be useful for correlating key genes and pathways that change across lifespan. However, the cell composition of bulk-dissected regions can vary greatly during brain development and across regions, limiting our understanding of cell type-specific contributions to gene expression. Single cell RNA-seq (scRNA-seq) and single nuclei RNA-seq (snRNA-seq), while often lacking depth and resolution of gene expression, can provide neuronal type-specific transcriptome and spatial localization data (Kang et al., 2011; Nowakowski et al., 2017; Li et al., 2018; Zhu et al., 2018; Ramos et al., 2022; Ament et al., 2023). Recent studies have demonstrated that mismatches of proteomic data when superimposed on transcriptomic data can occur, demonstrating post-transcriptional regulation (Carlyle et al., 2017; Breen et al., 2018). This has supported the need to generate human proteomics data across aging. One method of addressing this knowledge gap is through tissue proteomics as seen in studies on postmortem tissue, spanning from gestational to adult brain donors (Carlyle et al., 2017; Djuric et al., 2017; Pabba et al., 2017; Breen et al., 2018; Wingo et al., 2019). Another method is through ribosome profiling followed by sequencing (Ribo-seq), which provides insight on the translational regulation that leads to proteome diversity across human lifespan (Duffy et al., 2022).

Transcription can be regulated at the epigenetic level through controlling DNA accessibility. It is well known that during development and aging, the chromatin landscape can reflect and control gene regulation (Ziffra et al., 2021; Ament et al., 2023). DNA methylation is one aspect of epigenetic regulation and has been studied in the human brain across development, aging, and in diseased states (Jaffe et al., 2016; Li et al., 2018). Chromatin accessibility can also be examined through assay for transposase-accessible chromatin sequencing (ATAC-seq). A recent study performing single cell ATAC-seq of the developing human forebrain revealed cell-type and region-specific changes during corticogenesis (Ziffra et al., 2021). Further, rapid advancements in sequencing technology have led scientists to collaborate in generating multi-omics datasets, integrating various types of transcriptomic data with epigenomic data to provide a comprehensive molecular view of human brain development and aging (Li et al., 2018; Ament et al., 2023). By superimposing epigenomic datasets on transcriptomic datasets, it is possible to identify meaningful gene clusters that are similarly regulated epigenetically, potentially revealing developmental changes in DNA accessibility and master drivers of transcriptional programs in human neurons.

All the datasets described above have been used primarily to understand neurodevelopmental disorders, however, they are also suitable for mining developmentally-regulated genes for human axon growth and regeneration studies (Table 1).

**Table 1:** Human cortical sequencing datasets across lifespan.

Citation	Year	Ages	Region	Dataset
Johnson et al.	2009	18GW–23GW	Multiple brain regions	Microarray
Weickert et al.	2009	1MO–50YO	Prefrontal cortex	Microarray
Colantuoni et al.	2011	Fetal(−0.5)–78.23YO	Prefrontal cortex	Microarray
Kang et al.	2011	5.7PCW–82YO	Multiple brain regions	scRNA-seq
Pletikos et al.	2014	10 PCW–82YO	Multiple brain regions	Bulk RNA-seq
Jaffe et al.	2015	2nd trimester–50YO+	Dorsolateral prefrontal cortex	RNA-seq
Jaffe et al.	2016	14PCW–80YO	Dorsolateral prefrontal cortex	DNA methylation
Pabba et al.	2017	15YO–88YO	Orbitofrontal cortex, layer 2/3	Proteomics
Djuric et al.	2017	16GW–36GW	Multiple brain regions	Proteomics
Carlyle et al.	2017	Early infancy–42YO	Multiple brain regions	Proteomics*
Nowakowski et al.	2017	5.8PCW–37PCW	Multiple brain regions	scRNA-seq
Zhu et al.	2018	60PCD–11YO	Multiple brain regions	scRNA-seq
Li et al.	2018	5PCW–64YO	Multiple brain regions	DNA methylation, CHIP-seq, snRNA-seq, scRNA-seq, Bulk RNA-seq
Breen et al.	2018	1MO–49.5YO	Dorsolateral prefrontal cortex	Proteomics RNA-seq
Wingo et al.	2019	58.5YO–96.4YO	Multiple brain regions	Proteomics
Werling et al.	2020	6.14PCW–20YO	Dorsolateral prefrontal cortex	Bulk RNA-seq
Ziffra et al.	2021	18GW–21GW	Multiple brain regions	scATAC-seq
Duffy et al.	2022	12GW–82YO	Dorsolateral prefrontal cortex	Ribo-seq
Ramos et al.	2022	17GW–41GW	Multiple brain regions	snRNA-seq
Ament et al.	2023	4GW–68YO	Multiple brain regions	snRNA-seq, scRNA-seq, RNA-seq, scATAC-seq, snmC-seq2, Patch-seq

Compilation of epigenetic, transcriptomic, and proteomic age-span studies using either bulk or single cell human cortical tissue. PCW = post conception week, PCD = post conception day, GW = gestational week, YO = years old, MO = months old, ChIP-seq = chromatin immunoprecipitation sequencing, scRNA-seq = single cell RNA-seq, snRNA-seq = single nuclei RNA-seq, ribo-seq = ribosome profiling RNA-seq, snmC-seq2 = single cell methylation RNAseq, scATAC-seq = single cell chromatin accessibility RNA-seq, patch-seq = patch seq.

*same samples as those from BrainSpan (Kang et al., 2011).

## 4. Discussion: what do these human model systems mean for the future of axon regeneration studies?

The establishment of numerous techniques to make human CNS neurons allows us to ask species-specific questions about regulators of human axon growth. Each *in vitro* system has its unique positive attributes and limitations suitable for answering specific types of questions. While *in vitro* studies can provide a wealth of knowledge, the inability to study human neurons in an intact *in vivo* nervous system, which has a dynamic endogenous environment and age-specific signaling, cellular, and structural changes, has limited the adaptation of human model systems for studies of axon growth. However, developments in chimeric transplantation of human cells into the rodent CNS to dynamically visualize adult human axon growth in a systemic environment could take us one step closer to an improved human *in vivo* model. Harnessing the knowledge gained from these diverse human model systems and datasets may reveal novel human-specific, cell type-specific regulators of axon growth, as well as potentially identifying developmentally-regulated factors that could influence this growth. Ultimately, adding human systems to our studies of CNS axon growth and regeneration may accelerate and increase the success of the transition of preclinical studies to clinical trials.

### 4.1. Permission to reuse and copyright

Permission must be obtained for use of copyrighted material from other sources (including the web). Please note that it is compulsory to follow figure instructions.

## Author contributions

BL and DM co-designed, interpreted the relevant literature, and wrote the manuscript. All authors contributed to the article and approved the submitted version.

## Funding

The authors thank our funding sources: 1F30NS122478 (to BL), 1R21NS111192–01 (to DM), T32GM140935 (to University of Wisconsin-Madison Medical Scientist Training Program).

## Conflict of interest

The authors declare that the research was conducted in the absence of any commercial or financial relationships that could be construed as a potential conflict of interest.

## Publisher’s note

All claims expressed in this article are solely those of the authors and do not necessarily represent those of their affiliated organizations, or those of the publisher, the editors and the reviewers. Any product that may be evaluated in this article, or claim that may be made by its manufacturer, is not guaranteed or endorsed by the publisher.

## References

[ref1] Abernathy D. G. Kim W. K. McCoy M. J. Lake A. M. Ouwenga R. Lee S. W.. (2017). MicroRNAs induce a permissive chromatin environment that enables neuronal subtype-specific reprogramming of adult human fibroblasts. Cell Stem Cell 21:e9, 332–348.e9. doi: 10.1016/j.stem.2017.08.002, PMID: 28886366 PMC5679239

[ref2] Ambasudhan R. Talantova M. Coleman R. Yuan X. Zhu S. Lipton S. A.. (2011). Direct reprogramming of adult human fibroblasts to functional neurons under defined conditions. Cell Stem Cell 9, 113–118. doi: 10.1016/j.stem.2011.07.002, PMID: 21802386 PMC4567246

[ref3] Ament S. A. Adkins R. S. Carter R. Chrysostomou E. Colantuoni C. Crabtree J.. (2023). The neuroscience multi-Omic archive: a BRAIN initiative resource for single-cell transcriptomic and epigenomic data from the mammalian BRAIN. Nucleic Acids Res. 51, D1075–D1085. doi: 10.1093/nar/gkac962, PMID: 36318260 PMC9825473

[ref4] Andersen J. Revah O. Miura Y. Thom N. Amin N. D. Kelley K. W.. (2020). Generation of functional human 3D Cortico-motor Assembloids. Cells 183:e26 10.1016/j.cell.2020.11.017 PMC8711252 33333020

[ref5] Apara A. Galvao J. Wang Y. Blackmore M. Trillo A. Iwao K.. (2017). KLF9 and JNK3 interact to suppress axon regeneration in the adult CNS. J. Neurosci. 37, 9632–9644. doi: 10.1523/JNEUROSCI.0643-16.2017, PMID: 28871032 PMC5628408

[ref6] Arlotta P. Molyneaux B. J. Chen J. Inoue J. Kominami R. Macklis J. D. (2005). Neuronal subtype-specific genes that control corticospinal motor neuron development in vivo. Neuron 45, 207–221. doi: 10.1016/j.neuron.2004.12.036, PMID: 15664173

[ref7] Avila-Mendoza J. Subramani A. Denver R. J. (2020). Kruppel-like factors 9 and 13 Block axon growth by transcriptional repression of key components of the cAMP Signaling pathway. Front. Mol. Neurosci. 13:602638. doi: 10.3389/fnmol.2020.602638, PMID: 33281552 PMC7689098

[ref8] Aydin B. Kakumanu A. Rossillo M. Moreno-Estelles M. Garipler G. Ringstad N.. (2019). Proneural factors Ascl1 and Neurog2 contribute to neuronal subtype identities by establishing distinct chromatin landscapes. Nat. Neurosci. 22, 897–908. doi: 10.1038/s41593-019-0399-y, PMID: 31086315 PMC6556771

[ref9] Bandtlow C. E. Loschinger J. (1997). Developmental changes in neuronal responsiveness to the CNS myelin-associated neurite growth inhibitor NI-35/250. Eur. J. Neurosci. 9, 2743–2752. doi: 10.1111/j.1460-9568.1997.tb01703.x, PMID: 9517479

[ref10] Becker C. G. Becker T. (2002). Repellent guidance of regenerating optic axons by chondroitin sulfate glycosaminoglycans in zebrafish. J. Neurosci. 22, 842–853. doi: 10.1523/JNEUROSCI.22-03-00842.2002, PMID: 11826114 PMC6758477

[ref11] Bennett J. Emmady P. D. (2023). Spinal cord injuries. StatPearls. Treasure Island (FL). 32809556

[ref12] Blackmore M. Letourneau P. C. (2006). Changes within maturing neurons limit axonal regeneration in the developing spinal cord. J. Neurobiol. 66, 348–360. doi: 10.1002/neu.20224, PMID: 16408302

[ref13] Blackmore M. G. Wang Z. Lerch J. K. Motti D. Zhang Y. P. Shields C. B.. (2012). Kruppel-like factor 7 engineered for transcriptional activation promotes axon regeneration in the adult corticospinal tract. Proc. Natl. Acad. Sci. U. S. A. 109, 7517–7522. doi: 10.1073/pnas.1120684109, PMID: 22529377 PMC3358880

[ref14] Breen M. S. Ozcan S. Ramsey J. M. Wang Z. Ma’ayan A. Rustogi N.. (2018). Temporal proteomic profiling of postnatal human cortical development. Transl. Psychiatry 8:267. doi: 10.1038/s41398-018-0306-4, PMID: 30518843 PMC6281671

[ref15] Bregman B. S. Kunkel-Bagden E. McAtee M. O’Neill A. (1989). Extension of the critical period for developmental plasticity of the corticospinal pathway. J. Comp. Neurol. 282, 355–370. doi: 10.1002/cne.902820304, PMID: 2715387

[ref16] Cai D. Deng K. Mellado W. Lee J. Ratan R. R. Filbin M. T. (2002). Arginase I and polyamines act downstream from cyclic AMP in overcoming inhibition of axonal growth MAG and myelin in vitro. Neuron 35, 711–719. doi: 10.1016/S0896-6273(02)00826-7, PMID: 12194870

[ref17] Caiazzo M. Dell’Anno M. T. Dvoretskova E. Lazarevic D. Taverna S. Leo D.. (2011). Direct generation of functional dopaminergic neurons from mouse and human fibroblasts. Nature 476, 224–227. doi: 10.1038/nature10284, PMID: 21725324

[ref18] Cantuti-Castelvetri L. Fitzner D. Bosch-Queralt M. Weil M. T. Su M. Sen P.. (2018). Defective cholesterol clearance limits remyelination in the aged central nervous system. Science 359, 684–688. doi: 10.1126/science.aan4183, PMID: 29301957

[ref19] Cao Z. Gao Y. Bryson J. B. Hou J. Chaudhry N. Siddiq M.. (2006). The cytokine interleukin-6 is sufficient but not necessary to mimic the peripheral conditioning lesion effect on axonal growth. J. Neurosci. 26, 5565–5573. doi: 10.1523/JNEUROSCI.0815-06.2006, PMID: 16707807 PMC6675293

[ref20] Carlyle B. C. Kitchen R. R. Kanyo J. E. Voss E. Z. Pletikos M. Sousa A. M. M.. (2017). A multiregional proteomic survey of the postnatal human brain. Nat. Neurosci. 20, 1787–1795. doi: 10.1038/s41593-017-0011-2, PMID: 29184206 PMC5894337

[ref21] Cates K. McCoy M. J. Kwon J. S. Liu Y. Abernathy D. G. Zhang B.. (2021). Deconstructing stepwise fate conversion of human fibroblasts to neurons by MicroRNAs. Cell Stem Cell 28:e9, 127–140.e9. doi: 10.1016/j.stem.2020.08.015, PMID: 32961143 PMC7796891

[ref22] Chakraborty S. Ji H. Kabadi A. M. Gersbach C. A. Christoforou N. Leong K. W. (2014). A CRISPR/Cas9-based system for reprogramming cell lineage specification. Stem Cell Rep. 3, 940–947. doi: 10.1016/j.stemcr.2014.09.013, PMID: 25448066 PMC4264059

[ref23] Chavez A. Scheiman J. Vora S. Pruitt B. W. Tuttle M. P R Iyer E.. (2015). Highly efficient Cas9-mediated transcriptional programming. Nat. Methods 12, 326–328. doi: 10.1038/nmeth.3312, PMID: 25730490 PMC4393883

[ref24] Chen D. F. Jhaveri S. Schneider G. E. (1995). Intrinsic changes in developing retinal neurons result in regenerative failure of their axons. Proc. Natl. Acad. Sci. U. S. A. 92, 7287–7291. doi: 10.1073/pnas.92.16.7287, PMID: 7638182 PMC41324

[ref25] Chen S. Zhang J. Zhang D. Jiao J. (2019). Acquisition of functional neurons by direct conversion: switching the developmental clock directly. J. Genet. Genomics 46, 459–465. doi: 10.1016/j.jgg.2019.10.003, PMID: 31771824

[ref26] Cheng L. Gao L. Guan W. Mao J. Hu W. Qiu B.. (2015). Direct conversion of astrocytes into neuronal cells by drug cocktail. Cell Res. 25, 1269–1272. doi: 10.1038/cr.2015.120, PMID: 26427716 PMC4650423

[ref27] Chuang J. H. Tung L. C. Lin Y. (2015). Neural differentiation from embryonic stem cells in vitro: An overview of the signaling pathways. World J. Stem Cells 7, 437–447. doi: 10.4252/wjsc.v7.i2.437, PMID: 25815127 PMC4369499

[ref28] Cizkova D. Kakinohana O. Kucharova K. Marsala S. Johe K. Hazel T.. (2007). Functional recovery in rats with ischemic paraplegia after spinal grafting of human spinal stem cells. Neuroscience 147, 546–560. doi: 10.1016/j.neuroscience.2007.02.065, PMID: 17524565 PMC3417127

[ref29] Colantuoni C. Lipska B. K. Ye T. Hyde T. M. Tao R. Leek J. T.. (2011). Temporal dynamics and genetic control of transcription in the human prefrontal cortex. Nature 478, 519–523. doi: 10.1038/nature10524, PMID: 22031444 PMC3510670

[ref30] Curcio M. Bradke F. (2018). Axon regeneration in the central nervous system: facing the challenges from the inside. Annu. Rev. Cell Dev. Biol. 34, 495–521. doi: 10.1146/annurev-cellbio-100617-062508, PMID: 30044649

[ref31] Dai P. Harada Y. Takamatsu T. (2015). Highly efficient direct conversion of human fibroblasts to neuronal cells by chemical compounds. J. Clin. Biochem. Nutr. 56, 166–170. doi: 10.3164/jcbn.15-39, PMID: 26060345 PMC4454078

[ref32] de Lima S. Koriyama Y. Kurimoto T. Oliveira J. T. Yin Y. Li Y.. (2012). Full-length axon regeneration in the adult mouse optic nerve and partial recovery of simple visual behaviors. Proc. Natl. Acad. Sci. U. S. A. 109, 9149–9154. doi: 10.1073/pnas.1119449109, PMID: 22615390 PMC3384191

[ref33] Deng K. He H. Qiu J. Lorber B. Bryson J. B. Filbin M. T. (2009). Increased synthesis of spermidine as a result of upregulation of arginase I promotes axonal regeneration in culture and in vivo. J. Neurosci. 29, 9545–9552. doi: 10.1523/JNEUROSCI.1175-09.2009, PMID: 19641117 PMC6666538

[ref34] Dietz V. A. Roberts N. Knox K. Moore S. Pitonak M. Barr C.. (2022). Fighting for recovery on multiple fronts: the past, present, and future of clinical trials for spinal cord injury. Front. Cell. Neurosci. 16:977679. doi: 10.3389/fncel.2022.977679, PMID: 36212690 PMC9533868

[ref35] Djuric U. Rodrigues D. C. Batruch I. Ellis J. Shannon P. Diamandis P. (2017). Spatiotemporal proteomic profiling of human cerebral development. Mol. Cell. Proteomics 16, 1548–1562. doi: 10.1074/mcp.M116.066274, PMID: 28687556 PMC5587857

[ref36] Du K. Zheng S. Zhang Q. Li S. Gao X. Wang J.. (2015). Pten deletion promotes regrowth of corticospinal tract axons 1 year after spinal cord injury. J. Neurosci. 35, 9754–9763. doi: 10.1523/JNEUROSCI.3637-14.2015, PMID: 26134657 PMC6605149

[ref37] Duffy E. E. Finander B. Choi G. Carter A. C. Pritisanac I. Alam A.. (2022). Developmental dynamics of RNA translation in the human brain. Nat. Neurosci. 25, 1353–1365. doi: 10.1038/s41593-022-01164-9, PMID: 36171426 PMC10198132

[ref38] Dulin J. N. Adler A. F. Kumamaru H. Poplawski G. H. D. Lee-Kubli C. Strobl H.. (2018). Injured adult motor and sensory axons regenerate into appropriate organotypic domains of neural progenitor grafts. Nat. Commun. 9:84. doi: 10.1038/s41467-017-02613-x, PMID: 29311559 PMC5758751

[ref39] Dusart I. Airaksinen M. S. Sotelo C. (1997). Purkinje cell survival and axonal regeneration are age dependent: an *in vitro* study. J. Neurosci. 17, 3710–3726. doi: 10.1523/JNEUROSCI.17-10-03710.1997, PMID: 9133392 PMC6573677

[ref40] El Wazan L. Urrutia-Cabrera D. Wong R. C. (2019). Using transcription factors for direct reprogramming of neurons in vitro. World J. Stem Cells 11, 431–444. doi: 10.4252/wjsc.v11.i7.431, PMID: 31396370 PMC6682505

[ref41] Falkner S. Grade S. Dimou L. Conzelmann K. K. Bonhoeffer T. Gotz M.. (2016). Transplanted embryonic neurons integrate into adult neocortical circuits. Nature 539, 248–253. doi: 10.1038/nature20113, PMID: 27783592

[ref42] Fawcett J. W. (1997). Astrocytic and neuronal factors affecting axon regeneration in the damaged central nervous system. Cell Tissue Res. 290, 371–377. doi: 10.1007/s004410050943, PMID: 9321700

[ref43] Fiesel F. C. Ando M. Hudec R. Hill A. R. Castanedes-Casey M. Caulfield T. R.. (2015). (Patho-)physiological relevance of PINK1-dependent ubiquitin phosphorylation. EMBO Rep. 16, 1114–1130. doi: 10.15252/embr.201540514, PMID: 26162776 PMC4576981

[ref44] Filbin M. T. (2003). Myelin-associated inhibitors of axonal regeneration in the adult mammalian CNS. Nat. Rev. Neurosci. 4, 703–713. doi: 10.1038/nrn1195, PMID: 12951563

[ref45] Foran D. R. Peterson A. C. (1992). Myelin acquisition in the central nervous system of the mouse revealed by an MBP-lac Z transgene. J. Neurosci. 12, 4890–4897. doi: 10.1523/JNEUROSCI.12-12-04890.1992, PMID: 1281497 PMC6575777

[ref46] Fricker-Gates R. A. Shin J. J. Tai C. C. Catapano L. A. Macklis J. D. (2002). Late-stage immature neocortical neurons reconstruct interhemispheric connections and form synaptic contacts with increased efficiency in adult mouse cortex undergoing targeted neurodegeneration. J. Neurosci. 22, 4045–4056. doi: 10.1523/JNEUROSCI.22-10-04045.2002, PMID: 12019324 PMC6757654

[ref47] Gaillard A. Prestoz L. Dumartin B. Cantereau A. Morel F. Roger M.. (2007). Reestablishment of damaged adult motor pathways by grafted embryonic cortical neurons. Nat. Neurosci. 10, 1294–1299. doi: 10.1038/nn1970, PMID: 17828256

[ref48] Gaillard F. Sauve Y. (2007). Cell-based therapy for retina degeneration: the promise of a cure. Vis. Res. 47, 2815–2824. doi: 10.1016/j.visres.2007.06.018, PMID: 17719072

[ref49] Galvao J. Iwao K. Apara A. Wang Y. Ashouri M. Shah T. N.. (2018). The Kruppel-like factor gene target Dusp14 regulates axon growth and regeneration. Invest. Ophthalmol. Vis. Sci. 59, 2736–2747. doi: 10.1167/iovs.17-23319, PMID: 29860460 PMC5983061

[ref50] Gao Y. Deng K. Hou J. Bryson J. B. Barco A. Nikulina E.. (2004). Activated CREB is sufficient to overcome inhibitors in myelin and promote spinal axon regeneration in vivo. Neuron 44, 609–621. doi: 10.1016/j.neuron.2004.10.030, PMID: 15541310

[ref51] Gao L. Guan W. Wang M. Wang H. Yu J. Liu Q.. (2017). Direct generation of human neuronal cells from adult astrocytes by small molecules. Stem Cell Rep. 8, 538–547. doi: 10.1016/j.stemcr.2017.01.014, PMID: 28216149 PMC5355633

[ref52] Gaub P. Joshi Y. Wuttke A. Naumann U. Schnichels S. Heiduschka P.. (2011). The histone acetyltransferase p300 promotes intrinsic axonal regeneration. Brain 134, 2134–2148. doi: 10.1093/brain/awr142, PMID: 21705428

[ref53] Gaub P. Tedeschi A. Puttagunta R. Nguyen T. Schmandke A. Di Giovanni S. (2010). HDAC inhibition promotes neuronal outgrowth and counteracts growth cone collapse through CBP/p300 and P/CAF-dependent p53 acetylation. Cell Death Differ. 17, 1392–1408. doi: 10.1038/cdd.2009.216, PMID: 20094059

[ref54] Genovese T. Mazzon E. Di Paola R. Crisafulli C. Muia C. Bramanti P.. (2006). Increased oxidative-related mechanisms in the spinal cord injury in old rats. Neurosci. Lett. 393, 141–146. doi: 10.1016/j.neulet.2005.09.060, PMID: 16236449

[ref55] Geoffroy C. G. Hilton B. J. Tetzlaff W. Zheng B. (2016). Evidence for an age-dependent decline in axon regeneration in the adult mammalian central nervous system. Cell Rep. 15, 238–246. doi: 10.1016/j.celrep.2016.03.028, PMID: 27050519 PMC5050004

[ref56] Geoffroy C. G. Lorenzana A. O. Kwan J. P. Lin K. Ghassemi O. Ma A.. (2015). Effects of PTEN and Nogo Codeletion on corticospinal axon sprouting and regeneration in mice. J. Neurosci. 35, 6413–6428. doi: 10.1523/JNEUROSCI.4013-14.2015, PMID: 25904793 PMC4405557

[ref57] Geoffroy C. G. Meves J. M. Zheng B. (2017). The age factor in axonal repair after spinal cord injury: a focus on neuron-intrinsic mechanisms. Neurosci. Lett. 652, 41–49. doi: 10.1016/j.neulet.2016.11.003, PMID: 27818358 PMC5415436

[ref400] Giandomenico S. L. Mierau S. B. Gibbons G. M (2019). Cerebral organoids at the air–liquid interface generate diverse nerve tracts with functional output. Nat Neurosci, 22, 669–679. doi: 10.1038/s41593-019-0350-2, PMID: 30886407 PMC6436729

[ref58] Goldberg J. L. Klassen M. P. Hua Y. Barres B. A. (2002). Amacrine-signaled loss of intrinsic axon growth ability by retinal ganglion cells. Science 296, 1860–1864. doi: 10.1126/science.1068428, PMID: 12052959

[ref59] Gwak Y. S. Hains B. C. Johnson K. M. Hulsebosch C. E. (2004). Effect of age at time of spinal cord injury on behavioral outcomes in rat. J. Neurotrauma 21, 983–993. doi: 10.1089/0897715041650999, PMID: 15318998

[ref60] Hancock M. K. Kopp L. Kaur N. Hanson B. J. (2015). A facile method for simultaneously measuring neuronal cell viability and neurite outgrowth. Curr. Chem. Genom. Trans. Med. 9, 6–16. doi: 10.2174/2213988501509010006, PMID: 25853055 PMC4382562

[ref61] Horvath S. (2013). DNA methylation age of human tissues and cell types. Genome Biol. 14:R115. doi: 10.1186/gb-2013-14-10-r115, PMID: 24138928 PMC4015143

[ref62] Huang H. Miao L. Yang L. Liang F. Wang Q. Zhuang P.. (2019). AKT-dependent and -independent pathways mediate PTEN deletion-induced CNS axon regeneration. Cell Death Dis. 10:203. doi: 10.1038/s41419-018-1289-z, PMID: 30814515 PMC6393504

[ref63] Huh C. J. Zhang B. Victor M. B. Dahiya S. Batista L. F. Horvath S.. (2016). Maintenance of age in human neurons generated by microRNA-based neuronal conversion of fibroblasts. elife 5. doi: 10.7554/eLife.18648, PMID: 27644593 PMC5067114

[ref64] Jaffe A. E. Gao Y. Deep-Soboslay A. Tao R. Hyde T. M. Weinberger D. R.. (2016). Mapping DNA methylation across development, genotype and schizophrenia in the human frontal cortex. Nat. Neurosci. 19, 40–47. doi: 10.1038/nn.4181, PMID: 26619358 PMC4783176

[ref65] Jaffe A. E. Shin J. Collado-Torres L. Leek J. T. Tao R. Li C.. (2015). Developmental regulation of human cortex transcription and its clinical relevance at single base resolution. Nat. Neurosci. 18, 154–161. doi: 10.1038/nn.3898, PMID: 25501035 PMC4281298

[ref66] Jang Y. Choi J. Park N. Kang J. Kim M. Kim Y.. (2019). Development of immunocompatible pluripotent stem cells via CRISPR-based human leukocyte antigen engineering. Exp. Mol. Med. 51, 1–11. doi: 10.1038/s12276-018-0190-2, PMID: 30617277 PMC6323054

[ref67] Jin D. Liu Y. Sun F. Wang X. Liu X. He Z. (2015). Restoration of skilled locomotion by sprouting corticospinal axons induced by co-deletion of PTEN and SOCS3. Nat. Commun. 6:8074. doi: 10.1038/ncomms9074, PMID: 26598325 PMC4662086

[ref68] Johnson M. B. Kawasawa Y. I. Mason C. E. Krsnik Z. Coppola G. Bogdanovic D.. (2009). Functional and evolutionary insights into human brain development through global transcriptome analysis. Neuron 62, 494–509. doi: 10.1016/j.neuron.2009.03.027, PMID: 19477152 PMC2739738

[ref69] Jones L. L. Sajed D. Tuszynski M. H. (2003). Axonal regeneration through regions of chondroitin sulfate proteoglycan deposition after spinal cord injury: a balance of permissiveness and inhibition. J. Neurosci. 23, 9276–9288. doi: 10.1523/JNEUROSCI.23-28-09276.2003, PMID: 14561854 PMC6740563

[ref70] Kadoya K. Lu P. Nguyen K. Lee-Kubli C. Kumamaru H. Yao L.. (2016). Spinal cord reconstitution with homologous neural grafts enables robust corticospinal regeneration. Nat. Med. 22, 479–487. doi: 10.1038/nm.4066, PMID: 27019328 PMC4860037

[ref71] Kang H. J. Kawasawa Y. I. Cheng F. Zhu Y. Xu X. Li M.. (2011). Spatio-temporal transcriptome of the human brain. Nature 478, 483–489. doi: 10.1038/nature10523, PMID: 22031440 PMC3566780

[ref72] Keller G. Snodgrass H. R. (1999). Human embryonic stem cells: the future is now. Nat. Med. 5, 151–152. doi: 10.1038/5512, PMID: 9930859

[ref73] Kim J. Y. Nam Y. Rim Y. A. Ju J. H. (2022). Review of the current trends in clinical trials involving induced pluripotent stem cells. Stem Cell Rev. Rep. 18, 142–154. doi: 10.1007/s12015-021-10262-3, PMID: 34532844 PMC8445612

[ref74] Kim K. M. Thaqi M. Peterson D. A. Marr R. A. (2021). Induced neurons for disease Modeling and repair: a focus on non-fibroblastic cell sources in direct reprogramming. Front. Bioeng. Biotechnol. 9:658498. doi: 10.3389/fbioe.2021.658498, PMID: 33777923 PMC7995206

[ref75] Kitagawa T. Nagoshi N. Kamata Y. Kawai M. Ago K. Kajikawa K.. (2022). Modulation by DREADD reveals the therapeutic effect of human iPSC-derived neuronal activity on functional recovery after spinal cord injury. Stem Cell Reports 17, 127–142. doi: 10.1016/j.stemcr.2021.12.005, PMID: 35021049 PMC8758967

[ref76] Kramer A. S. Harvey A. R. Plant G. W. Hodgetts S. I. (2013). Systematic review of induced pluripotent stem cell technology as a potential clinical therapy for spinal cord injury. Cell Transplant. 22, 571–617. doi: 10.3727/096368912X655208, PMID: 22944020

[ref77] Kramer A. A. Olson G. M. Chakraborty A. Blackmore M. G. (2021). Promotion of corticospinal tract growth by KLF6 requires an injury stimulus and occurs within four weeks of treatment. Exp. Neurol. 339:113644. doi: 10.1016/j.expneurol.2021.113644, PMID: 33592210 PMC8224817

[ref78] Kumamaru H. Kadoya K. Adler A. F. Takashima Y. Graham L. Coppola G.. (2018a). Generation and post-injury integration of human spinal cord neural stem cells. Nat. Methods 15, 723–731. doi: 10.1038/s41592-018-0074-3, PMID: 30082899

[ref79] Kumamaru H. Lu P. Rosenzweig E. S. Tuszynski M. H. (2018b). Activation of intrinsic growth State enhances host axonal regeneration into neural progenitor cell grafts. Stem Cell Rep. 11, 861–868. doi: 10.1016/j.stemcr.2018.08.009, PMID: 30197116 PMC6178188

[ref80] Kurimoto T. Yin Y. Omura K. Gilbert H. Y. Kim D. Cen L. P.. (2010). Long-distance axon regeneration in the mature optic nerve: contributions of oncomodulin, cAMP, and pten gene deletion. J. Neurosci. 30, 15654–15663. doi: 10.1523/JNEUROSCI.4340-10.2010, PMID: 21084621 PMC3001271

[ref81] Lam H. J. Patel S. Wang A. Chu J. Li S. (2010). *In vitro* regulation of neural differentiation and axon growth by growth factors and bioactive nanofibers. Tissue Eng. Part A 16, 2641–2648. doi: 10.1089/ten.tea.2009.0414, PMID: 20367289 PMC2947452

[ref82] Lerch J. K. Martinez-Ondaro Y. R. Bixby J. L. Lemmon V. P. (2014). cJun promotes CNS axon growth. Mol. Cell. Neurosci. 59, 97–105. doi: 10.1016/j.mcn.2014.02.002, PMID: 24521823 PMC4008678

[ref83] Lewandowski G. Steward O. (2014). AAVshRNA-mediated suppression of PTEN in adult rats in combination with salmon fibrin administration enables regenerative growth of corticospinal axons and enhances recovery of voluntary motor function after cervical spinal cord injury. J. Neurosci. 34, 9951–9962. doi: 10.1523/JNEUROSCI.1996-14.2014, PMID: 25057197 PMC4107411

[ref84] Li H. Chen G. (2016). In vivo reprogramming for CNS repair: regenerating neurons from endogenous glial cells. Neuron 91, 728–738. doi: 10.1016/j.neuron.2016.08.004, PMID: 27537482 PMC5466364

[ref85] Li X. Li M. Tian L. Chen J. Liu R. Ning B. (2020). Reactive Astrogliosis: implications in spinal cord injury progression and therapy. Oxidative Med. Cell. Longev. 2020, 1–14. doi: 10.1155/2020/9494352 PMC7455824 32884625

[ref86] Li M. Santpere G. Imamura Kawasawa Y. Evgrafov O. V. Gulden F. O. Pochareddy S.. (2018). Integrative functional genomic analysis of human brain development and neuropsychiatric risks. Science 362. doi: 10.1126/science.aat7615, PMID: 30545854 PMC6413317

[ref87] Li X. Zuo X. Jing J. Ma Y. Wang J. Liu D.. (2015). Small-molecule-driven direct reprogramming of mouse fibroblasts into functional neurons. Cell Stem Cell 17, 195–203. doi: 10.1016/j.stem.2015.06.003, PMID: 26253201

[ref88] Lim S. M. Choi W. J. Oh K. W. Xue Y. Choi J. Y. Kim S. H.. (2016). Directly converted patient-specific induced neurons mirror the neuropathology of FUS with disrupted nuclear localization in amyotrophic lateral sclerosis. Mol. Neurodegener. 11:8. doi: 10.1186/s13024-016-0075-6, PMID: 26795035 PMC4722778

[ref89] Lim J. H. Stafford B. K. Nguyen P. L. Lien B. V. Wang C. Zukor K.. (2016). Neural activity promotes long-distance, target-specific regeneration of adult retinal axons. Nat. Neurosci. 19, 1073–1084. 27399843 10.1038/nn.4340 PMC5708130

[ref90] Liu L. Huang J. S. Han C. Zhang G. X. Xu X. Y. Shen Y.. (2016). Induced pluripotent stem cells in Huntington’s disease: disease Modeling and the potential for cell-based therapy. Mol. Neurobiol. 53, 6698–6708. doi: 10.1007/s12035-015-9601-8, PMID: 26659595 PMC5317136

[ref91] Liu K. Lu Y. Lee J. K. Samara R. Willenberg R. Sears-Kraxberger I.. (2010). PTEN deletion enhances the regenerative ability of adult corticospinal neurons. Nat. Neurosci. 13, 1075–1081. doi: 10.1038/nn.2603, PMID: 20694004 PMC2928871

[ref92] Liu Y. Xue Y. Ridley S. Zhang D. Rezvani K. Fu X. D.. (2014). Direct reprogramming of Huntington’s disease patient fibroblasts into neuron-like cells leads to abnormal neurite outgrowth, increased cell death, and aggregate formation. PLoS One 9:e109621. doi: 10.1371/journal.pone.0109621, PMID: 25275533 PMC4183653

[ref93] Liu M. L. Zang T. Zhang C. L. (2016). Direct lineage reprogramming reveals disease-specific phenotypes of motor neurons from human ALS patients. Cell Rep. 14, 115–128. doi: 10.1016/j.celrep.2015.12.018, PMID: 26725112 PMC4706770

[ref94] Liu M. L. Zang T. Zou Y. Chang J. C. Gibson J. R. Huber K. M.. (2013). Small molecules enable neurogenin 2 to efficiently convert human fibroblasts into cholinergic neurons. Nat. Commun. 4:2183. doi: 10.1038/ncomms3183, PMID: 23873306 PMC3843951

[ref95] Lu Y. Brommer B. Tian X. Krishnan A. Meer M. Wang C.. (2020). Reprogramming to recover youthful epigenetic information and restore vision. Nature 588, 124–129. doi: 10.1038/s41586-020-2975-4, PMID: 33268865 PMC7752134

[ref96] Lu P. Ceto S. Wang Y. Graham L. Wu D. Kumamaru H.. (2017). Prolonged human neural stem cell maturation supports recovery in injured rodent CNS. J. Clin. Invest. 127, 3287–3299. doi: 10.1172/JCI92955, PMID: 28825600 PMC5669577

[ref97] Lu P. Kadoya K. Tuszynski M. H. (2014a). Axonal growth and connectivity from neural stem cell grafts in models of spinal cord injury. Curr. Opin. Neurobiol. 27, 103–109. doi: 10.1016/j.conb.2014.03.010, PMID: 24709371

[ref98] Lu P. Wang Y. Graham L. McHale K. Gao M. Wu D.. (2012). Long-distance growth and connectivity of neural stem cells after severe spinal cord injury. Cells 150, 1264–1273. doi: 10.1016/j.cell.2012.08.020, PMID: 22980985 PMC3445432

[ref99] Lu P. Woodruff G. Wang Y. Graham L. Hunt M. Wu D.. (2014b). Long-distance axonal growth from human induced pluripotent stem cells after spinal cord injury. Neuron 83, 789–796. doi: 10.1016/j.neuron.2014.07.014, PMID: 25123310 PMC4144679

[ref100] Ma X. M. Blenis J. (2009). Molecular mechanisms of mTOR-mediated translational control. Nat. Rev. Mol. Cell Biol. 10, 307–318. doi: 10.1038/nrm2672, PMID: 19339977

[ref101] Mar F. M. Bonni A. Sousa M. M. (2014). Cell intrinsic control of axon regeneration. EMBO Rep. 15, 254–263. doi: 10.1002/embr.201337723, PMID: 24531721 PMC3989691

[ref102] McKeon R. J. Jurynec M. J. Buck C. R. (1999). The chondroitin sulfate proteoglycans neurocan and phosphacan are expressed by reactive astrocytes in the chronic CNS glial scar. J. Neurosci. 19, 10778–10788. doi: 10.1523/JNEUROSCI.19-24-10778.1999, PMID: 10594061 PMC6784959

[ref103] Mehta S. T. Luo X. Park K. K. Bixby J. L. Lemmon V. P. (2016). Hyperactivated Stat3 boosts axon regeneration in the CNS. Exp. Neurol. 280, 115–120. doi: 10.1016/j.expneurol.2016.03.004, PMID: 27060489 PMC4888791

[ref104] Mertens J. Herdy J. R. Traxler L. Schafer S. T. Schlachetzki J. C. M. Bohnke L.. (2021). Age-dependent instability of mature neuronal fate in induced neurons from Alzheimer’s patients. Cell Stem Cell 28, 1533–1548.e6. doi: 10.1016/j.stem.2021.04.004, PMID: 33910058 PMC8423435

[ref105] Mertens J. Paquola A. C. M. Ku M. Hatch E. Bohnke L. Ladjevardi S.. (2015). Directly reprogrammed human neurons retain aging-associated transcriptomic signatures and reveal age-related nucleocytoplasmic defects. Cell Stem Cell 17, 705–718. doi: 10.1016/j.stem.2015.09.001, PMID: 26456686 PMC5929130

[ref106] Mertens J. Reid D. Lau S. Kim Y. Gage F. H. (2018). Aging in a dish: iPSC-derived and directly induced neurons for studying brain aging and age-related neurodegenerative diseases. Annu. Rev. Genet. 52, 271–293. doi: 10.1146/annurev-genet-120417-031534, PMID: 30208291 PMC6415910

[ref107] Moore D. L. Blackmore M. G. Hu Y. Kaestner K. H. Bixby J. L. Lemmon V. P.. (2009). KLF family members regulate intrinsic axon regeneration ability. Science 326, 298–301. doi: 10.1126/science.1175737, PMID: 19815778 PMC2882032

[ref108] Muller F. De Virgiliis F. Kong G. Zhou L. Serger E. Chadwick J.. (2022). CBP/p300 activation promotes axon growth, sprouting, and synaptic plasticity in chronic experimental spinal cord injury with severe disability. PLoS Biol. 20:e3001310. doi: 10.1371/journal.pbio.3001310, PMID: 36126035 PMC9488786

[ref109] Nagoshi N. Okano H. (2017). Applications of induced pluripotent stem cell technologies in spinal cord injury. J. Neurochem. 141, 848–860. doi: 10.1111/jnc.13986, PMID: 28199003

[ref110] Nathan F. M. Ohtake Y. Wang S. Jiang X. Sami A. Guo H.. (2020). Upregulating Lin28a promotes axon regeneration in adult mice with optic nerve and spinal cord injury. Mol. Ther. 28, 1902–1917. doi: 10.1016/j.ymthe.2020.04.010, PMID: 32353321 PMC7403348

[ref111] Nori S. Okada Y. Yasuda A. Tsuji O. Takahashi Y. Kobayashi Y.. (2011). Grafted human-induced pluripotent stem-cell-derived neurospheres promote motor functional recovery after spinal cord injury in mice. Proc. Natl. Acad. Sci. U. S. A. 108, 16825–16830. doi: 10.1073/pnas.1108077108, PMID: 21949375 PMC3189018

[ref112] Nowakowski T. J. Bhaduri A. Pollen A. A. Alvarado B. Mostajo-Radji M. A. Di Lullo E.. (2017). Spatiotemporal gene expression trajectories reveal developmental hierarchies of the human cortex. Science 358, 1318–1323. doi: 10.1126/science.aap8809, PMID: 29217575 PMC5991609

[ref113] O’Donovan K. J. Ma K. Guo H. Wang C. Sun F. Han S. B.. (2014). B-RAF kinase drives developmental axon growth and promotes axon regeneration in the injured mature CNS. J. Exp. Med. 211, 801–814. doi: 10.1084/jem.20131780, PMID: 24733831 PMC4010899

[ref114] Oh Y. M. Lee S. W. Kim W. K. Chen S. Church V. A. Cates K.. (2022). Age-related Huntington’s disease progression modeled in directly reprogrammed patient-derived striatal neurons highlights impaired autophagy. Nat. Neurosci. 25, 1420–1433. doi: 10.1038/s41593-022-01185-4, PMID: 36303071 PMC10162007

[ref115] Ohtake Y. Park D. Abdul-Muneer P. M. Li H. Xu B. Sharma K.. (2014). The effect of systemic PTEN antagonist peptides on axon growth and functional recovery after spinal cord injury. Biomaterials 35, 4610–4626. doi: 10.1016/j.biomaterials.2014.02.037, PMID: 24630093 PMC4195449

[ref116] Okubo T. Nagoshi N. Kohyama J. Tsuji O. Shinozaki M. Shibata S.. (2018). Treatment with a gamma-secretase inhibitor promotes functional recovery in human iPSC- derived transplants for chronic spinal cord injury. Stem Cell Rep. 11, 1416–1432. doi: 10.1016/j.stemcr.2018.10.022, PMID: 30503258 PMC6294244

[ref117] Pabba M. Scifo E. Kapadia F. Nikolova Y. S. Ma T. Mechawar N.. (2017). Resilient protein co-expression network in male orbitofrontal cortex layer 2/3 during human aging. Neurobiol. Aging 58, 180–190. doi: 10.1016/j.neurobiolaging.2017.06.023, PMID: 28750307 PMC5581682

[ref118] Pang Z. P. Yang N. Vierbuchen T. Ostermeier A. Fuentes D. R. Yang T. Q.. (2011). Induction of human neuronal cells by defined transcription factors. Nature 476, 220–223. doi: 10.1038/nature10202, PMID: 21617644 PMC3159048

[ref119] Park K. K. Liu K. Hu Y. Kanter J. L. He Z. (2010). PTEN/mTOR and axon regeneration. Exp. Neurol. 223, 45–50. doi: 10.1016/j.expneurol.2009.12.032, PMID: 20079353

[ref120] Park K. K. Liu K. Hu Y. Smith P. D. Wang C. Cai B.. (2008). Promoting axon regeneration in the adult CNS by modulation of the PTEN/mTOR pathway. Science 322, 963–966. doi: 10.1126/science.1161566, PMID: 18988856 PMC2652400

[ref121] Perrin S. (2014). Preclinical research: make mouse studies work. Nature 507, 423–425. doi: 10.1038/507423a, PMID: 24678540

[ref122] Pfisterer U. Kirkeby A. Torper O. Wood J. Nelander J. Dufour A.. (2011). Direct conversion of human fibroblasts to dopaminergic neurons. Proc. Natl. Acad. Sci. U. S. A. 108, 10343–10348. doi: 10.1073/pnas.1105135108, PMID: 21646515 PMC3121829

[ref123] Piao J. Zabierowski S. Dubose B. N. Hill E. J. Navare M. Claros N.. (2021). Preclinical efficacy and safety of a human embryonic stem cell-derived midbrain dopamine progenitor product, MSK-DA01. Cell Stem Cell 28, 217–229.e7. doi: 10.1016/j.stem.2021.01.004, PMID: 33545080 PMC7903922

[ref124] Pita-Thomas W. Goncalves T. M. Kumar A. Zhao G. Cavalli V. (2021). Genome-wide chromatin accessibility analyses provide a map for enhancing optic nerve regeneration. Sci. Rep. 11:14924. doi: 10.1038/s41598-021-94341-y, PMID: 34290335 PMC8295311

[ref125] Pletikos M. Sousa A. M. Sedmak G. Meyer K. A. Zhu Y. Cheng F.. (2014). Temporal specification and bilaterality of human neocortical topographic gene expression. Neuron 81, 321–332. doi: 10.1016/j.neuron.2013.11.018, PMID: 24373884 PMC3931000

[ref126] Poplawski G. H. D. Kawaguchi R. Van Niekerk E. Lu P. Mehta N. Canete P.. (2020). Injured adult neurons regress to an embryonic transcriptional growth state. Nature 581, 77–82. doi: 10.1038/s41586-020-2200-5, PMID: 32376949

[ref127] Qin S. Zou Y. Zhang C. L. (2013). Cross-talk between KLF4 and STAT3 regulates axon regeneration. Nat. Commun. 4:2633. doi: 10.1038/ncomms3633, PMID: 24129709 PMC3867821

[ref128] Ramos S. I. Mussa Z. M. Falk E. N. Pai B. Giotti B. Allette K.. (2022). An atlas of late prenatal human neurodevelopment resolved by single-nucleus transcriptomics. Nat. Commun. 13:7671. doi: 10.1038/s41467-022-34975-2, PMID: 36509746 PMC9744747

[ref129] Redmond L. Kashani A. H. Ghosh A. (2002). Calcium regulation of dendritic growth via CaM kinase IV and CREB-mediated transcription. Neuron 34, 999–1010. doi: 10.1016/S0896-6273(02)00737-7, PMID: 12086646

[ref130] Rim Y. A. Park N. Nam Y. Ham D. S. Kim J. W. Ha H. Y.. (2018). Recent progress of national banking project on homozygous HLA-typed induced pluripotent stem cells in South Korea. J. Tissue Eng. Regen. Med. 12, e1531–e1536. doi: 10.1002/term.2578, PMID: 28941241

[ref131] Rosenzweig E. S. Brock J. H. Lu P. Kumamaru H. Salegio E. A. Kadoya K.. (2018). Restorative effects of human neural stem cell grafts on the primate spinal cord. Nat. Med. 24, 484–490. doi: 10.1038/nm.4502, PMID: 29480894 PMC5922761

[ref132] Safaiyan S. Kannaiyan N. Snaidero N. Brioschi S. Biber K. Yona S.. (2016). Age-related myelin degradation burdens the clearance function of microglia during aging. Nat. Neurosci. 19, 995–998. doi: 10.1038/nn.4325, PMID: 27294511 PMC7116794

[ref133] Savell K. E. Sultan F. A. Day J. J. (2019). A novel dual lentiviral CRISPR-based transcriptional activation system for gene expression regulation in neurons. Biol. Protoc. 9:e3348. doi: 10.21769/BioProtoc.3348, PMID: 33654850 PMC7854184

[ref134] Saxton R. A. Sabatini D. M. (2017). mTOR Signaling in growth, metabolism, and disease. Cells 169, 361–371. doi: 10.1016/j.cell.2017.03.035, PMID: 28388417

[ref135] Seo W. M. Yoon J. Lee J. H. Lee Y. Lee H. Geum D.. (2022). Modeling axonal regeneration by changing cytoskeletal dynamics in stem cell-derived motor nerve organoids. Sci. Rep. 12:2082. doi: 10.1038/s41598-022-05645-6, PMID: 35136073 PMC8827082

[ref136] Shamblott M. J. Axelman J. Wang S. Bugg E. M. Littlefield J. W. Donovan P. J.. (1998). Derivation of pluripotent stem cells from cultured human primordial germ cells. Proc. Natl. Acad. Sci. U. S. A. 95, 13726–13731. doi: 10.1073/pnas.95.23.13726, PMID: 9811868 PMC24887

[ref137] Sherman S. P. Bang A. G. (2018). High-throughput screen for compounds that modulate neurite growth of human induced pluripotent stem cell-derived neurons. Dis. Model. Mech. 11. doi: 10.1242/dmm.031906, PMID: 29361516 PMC5894944

[ref138] Shin H. S. Kim H. J. Min S. K. Kim S. H. Lee B. M. Jeon N. L. (2010). Compartmental culture of embryonic stem cell-derived neurons in microfluidic devices for use in axonal biology. Biotechnol. Lett. 32, 1063–1070. doi: 10.1007/s10529-010-0280-2, PMID: 20424889

[ref139] Siegenthaler M. M. Ammon D. L. Keirstead H. S. (2008). Myelin pathogenesis and functional deficits following SCI are age-associated. Exp. Neurol. 213, 363–371. doi: 10.1016/j.expneurol.2008.06.015, PMID: 18644369 PMC3445440

[ref140] Sirenko O. Hesley J. Rusyn I. Cromwell E. F. (2014). High-content high-throughput assays for characterizing the viability and morphology of human iPSC-derived neuronal cultures. Assay Drug Dev. Technol. 12, 536–547. doi: 10.1089/adt.2014.592, PMID: 25506803 PMC4270163

[ref141] Snow D. M. Lemmon V. Carrino D. A. Caplan A. I. Silver J. (1990). Sulfated proteoglycans in astroglial barriers inhibit neurite outgrowth in vitro. Exp. Neurol. 109, 111–130. doi: 10.1016/S0014-4886(05)80013-5, PMID: 2141574

[ref142] Son E. Y. Ichida J. K. Wainger B. J. Toma J. S. Rafuse V. F. Woolf C. J.. (2011). Conversion of mouse and human fibroblasts into functional spinal motor neurons. Cell Stem Cell 9, 205–218. doi: 10.1016/j.stem.2011.07.014, PMID: 21852222 PMC3188987

[ref143] Stewart A. N. Lowe J. L. Glaser E. P. Mott C. A. Shahidehpour R. K. McFarlane K. E.. (2021). Acute inflammatory profiles differ with sex and age after spinal cord injury. J. Neuroinflamm. 18:113. doi: 10.1186/s12974-021-02161-8, PMID: 33985529 PMC8120918

[ref144] Sugai K. Sumida M. Shofuda T. Yamaguchi R. Tamura T. Kohzuki T.. (2021). First-in-human clinical trial of transplantation of iPSC-derived NS/PCs in subacute complete spinal cord injury: study protocol. Regen. Ther. 18, 321–333. doi: 10.1016/j.reth.2021.08.005, PMID: 34522725 PMC8427225

[ref145] Sun F. Park K. K. Belin S. Wang D. Lu T. Chen G.. (2011). Sustained axon regeneration induced by co-deletion of PTEN and SOCS3. Nature 480, 372–375. doi: 10.1038/nature10594, PMID: 22056987 PMC3240702

[ref146] Takahashi K. Tanabe K. Ohnuki M. Narita M. Ichisaka T. Tomoda K.. (2007). Induction of pluripotent stem cells from adult human fibroblasts by defined factors. Cells 131, 861–872. doi: 10.1016/j.cell.2007.11.019, PMID: 18035408

[ref147] Tanabe K. Ang C. E. Chanda S. Olmos V. H. Haag D. Levinson D. F.. (2018). Transdifferentiation of human adult peripheral blood T cells into neurons. Proc. Natl. Acad. Sci. U. S. A. 115, 6470–6475. doi: 10.1073/pnas.1720273115, PMID: 29866841 PMC6016798

[ref148] Tang X. Davies J. E. Davies S. J. (2003). Changes in distribution, cell associations, and protein expression levels of NG2, neurocan, phosphacan, brevican, versican V2, and tenascin-C during acute to chronic maturation of spinal cord scar tissue. J. Neurosci. Res. 71, 427–444. doi: 10.1002/jnr.10523, PMID: 12526031

[ref149] Tang Y. Liu M. L. Zang T. Zhang C. L. (2017). Direct reprogramming rather than iPSC-based reprogramming maintains aging hallmarks in human motor neurons. Front. Mol. Neurosci. 10:359. doi: 10.3389/fnmol.2017.00359, PMID: 29163034 PMC5676779

[ref150] Tao Y. Vermilyea S. C. Zammit M. Lu J. Olsen M. Metzger J. M.. (2021). Autologous transplant therapy alleviates motor and depressive behaviors in parkinsonian monkeys. Nat. Med. 27, 632–639. doi: 10.1038/s41591-021-01257-1, PMID: 33649496 PMC8198752

[ref151] Tao Y. Zhang S. C. (2016). Neural subtype specification from human pluripotent stem cells. Cell Stem Cell 19, 573–586. doi: 10.1016/j.stem.2016.10.015, PMID: 27814479 PMC5127287

[ref152] Teotia P. Van Hook M. J. Fischer D. Ahmad I. (2019). Human retinal ganglion cell axon regeneration by recapitulating developmental mechanisms: effects of recruitment of the mTOR pathway. Development 146. doi: 10.1242/dev.178012, PMID: 31273087 PMC6633601

[ref153] Thomson J. A. Itskovitz-Eldor J. Shapiro S. S. Waknitz M. A. Swiergiel J. J.. (1998). Embryonic stem cell lines derived from human blastocysts. Science 282, 1145–1147. doi: 10.1126/science.282.5391.1145, PMID: 9804556

[ref154] Trakhtenberg E. F. Li Y. Feng Q. Tso J. Rosenberg P. A. Goldberg J. L.. (2018). Zinc chelation and Klf9 knockdown cooperatively promote axon regeneration after optic nerve injury. Exp. Neurol. 300, 22–29. doi: 10.1016/j.expneurol.2017.10.025, PMID: 29106981 PMC5745290

[ref155] Usvald D. Vodicka P. Hlucilova J. Prochazka R. Motlik J. Kuchorova K.. (2010). Analysis of dosing regimen and reproducibility of intraspinal grafting of human spinal stem cells in immunosuppressed minipigs. Cell Transplant. 19, 1103–1122. doi: 10.3727/096368910X503406, PMID: 20412634

[ref156] Vadodaria K. C. Mertens J. Paquola A. Bardy C. Li X. Jappelli R.. (2016). Generation of functional human serotonergic neurons from fibroblasts. Mol. Psychiatry 21, 49–61. doi: 10.1038/mp.2015.161, PMID: 26503761

[ref157] van Gorp S. Leerink M. Kakinohana O. Platoshyn O. Santucci C. Galik J.. (2013). Amelioration of motor/sensory dysfunction and spasticity in a rat model of acute lumbar spinal cord injury by human neural stem cell transplantation. Stem Cell Res Ther 4:57. doi: 10.1186/scrt209, PMID: 23710605 PMC3706882

[ref158] Vasan L. Park E. David L. A. Fleming T. Schuurmans C. (2021). Direct neuronal reprogramming: bridging the gap between basic science and clinical application. Front. Cell Dev. Biol. 9:681087. doi: 10.3389/fcell.2021.681087, PMID: 34291049 PMC8287587

[ref159] Venkatesh I. Mehra V. Wang Z. Califf B. Blackmore M. G. (2018). Developmental chromatin restriction of pro-growth gene networks acts as an epigenetic barrier to axon regeneration in cortical neurons. Dev. Neurobiol. 78, 960–977. doi: 10.1002/dneu.22605, PMID: 29786967 PMC6204296

[ref160] Venkatesh I. Simpson M. T. Coley D. M. Blackmore M. G. (2016). Epigenetic profiling reveals a developmental decrease in promoter accessibility during cortical maturation in vivo. Neuroepigenetics 8, 19–26. doi: 10.1016/j.nepig.2016.10.002, PMID: 27990351 PMC5159751

[ref161] Victor M. B. Richner M. Hermanstyne T. O. Ransdell J. L. Sobieski C. Deng P. Y.. (2014). Generation of human striatal neurons by microRNA-dependent direct conversion of fibroblasts. Neuron 84, 311–323. doi: 10.1016/j.neuron.2014.10.016, PMID: 25374357 PMC4223654

[ref162] Victor M. B. Richner M. Olsen H. E. Lee S. W. Monteys A. M. Ma C.. (2018). Striatal neurons directly converted from Huntington’s disease patient fibroblasts recapitulate age-associated disease phenotypes. Nat. Neurosci. 21, 341–352. doi: 10.1038/s41593-018-0075-7, PMID: 29403030 PMC5857213

[ref163] Wan X. Y. Xu L. Y. Li B. Sun Q. H. Ji Q. L. Huang D. D.. (2018). Chemical conversion of human lung fibroblasts into neuronal cells. Int. J. Mol. Med. 41, 1463–1468. doi: 10.3892/ijmm.2018.3375, PMID: 29328434 PMC5819915

[ref164] Wang J. He Q. Zhang K. Sun H. Zhang G. Liang H.. (2020). Quick commitment and efficient reprogramming route of direct induction of retinal ganglion cell-like neurons. Stem Cell Rep. 15, 1095–1110. doi: 10.1016/j.stemcr.2020.09.008, PMID: 33096050 PMC7663790

[ref165] Wang J. T. Kunzevitzky N. J. Dugas J. C. Cameron M. Barres B. A. Goldberg J. L. (2007). Disease gene candidates revealed by expression profiling of retinal ganglion cell development. J. Neurosci. 27, 8593–8603. doi: 10.1523/JNEUROSCI.4488-06.2007, PMID: 17687037 PMC2885852

[ref166] Wang Z. Mehra V. Simpson M. T. Maunze B. Chakraborty A. Holan L.. (2018). KLF6 and STAT3 co-occupy regulatory DNA and functionally synergize to promote axon growth in CNS neurons. Sci. Rep. 8:12565. doi: 10.1038/s41598-018-31101-5, PMID: 30135567 PMC6105645

[ref167] Wang H. Yang Y. Liu J. Qian L. (2021). Direct cell reprogramming: approaches, mechanisms and progress. Nat. Rev. Mol. Cell Biol. 22, 410–424. doi: 10.1038/s41580-021-00335-z, PMID: 33619373 PMC8161510

[ref168] Wang Y. K. Zhu W. W. Wu M. H. Wu Y. H. Liu Z. X. Liang L. M.. (2018). Human clinical-Grade parthenogenetic ESC-derived dopaminergic neurons recover locomotive defects of nonhuman primate models of Parkinson’s disease. Stem Cell Rep. 11, 171–182. doi: 10.1016/j.stemcr.2018.05.010, PMID: 29910127 PMC6067059

[ref169] Waxman S. G. Foster R. E. (1980). Development of the axon membrane during differentiation of myelinated fibres in spinal nerve roots. Proc. R. Soc. Lond. B Biol. Sci. 209, 441–446. doi: 10.1098/rspb.1980.0105, PMID: 6161376

[ref170] Weickert C. S. Elashoff M. Richards A. B. Sinclair D. Bahn S. Paabo S.. (2009). Transcriptome analysis of male-female differences in prefrontal cortical development. Mol. Psychiatry 14, 558–561. doi: 10.1038/mp.2009.5, PMID: 19455171

[ref171] Weng Y. L. Wang X. An R. Cassin J. Vissers C. Liu Y.. (2018). Epitranscriptomic m(6)a regulation of axon regeneration in the adult mammalian nervous system. Neuron 97:e6, 313–325.e6. doi: 10.1016/j.neuron.2017.12.036, PMID: 29346752 PMC5777326

[ref172] Werling D. M. Pochareddy S. Choi J. An J. Y. Sheppard B. Peng M.. (2020). Whole-genome and RNA sequencing reveal variation and transcriptomic coordination in the developing human prefrontal cortex. Cell Rep. 31:107489. doi: 10.1016/j.celrep.2020.03.053, PMID: 32268104 PMC7295160

[ref173] Wertheim L. Edri R. Goldshmit Y. Kagan T. Noor N. Ruban A.. (2022). Regenerating the injured spinal cord at the chronic phase by engineered iPSCs-derived 3D neuronal networks. Adv. Sci. (Weinh) 9:e2105694. doi: 10.1002/advs.202105694, PMID: 35128819 PMC9008789

[ref174] Wingo A. P. Dammer E. B. Breen M. S. Logsdon B. A. Duong D. M. Troncosco J. C.. (2019). Large-scale proteomic analysis of human brain identifies proteins associated with cognitive trajectory in advanced age. Nat. Commun. 10:1619. doi: 10.1038/s41467-019-09613-z, PMID: 30962425 PMC6453881

[ref175] Xie L. Cen L. P. Li Y. Gilbert H. Y. Strelko O. Berlinicke C.. (2022). Monocyte-derived SDF1 supports optic nerve regeneration and alters retinal ganglion cells’ response to Pten deletion. Proc. Natl. Acad. Sci. U. S. A. 119:e2113751119. doi: 10.1073/pnas.2113751119, PMID: 35394873 PMC9169637

[ref176] Xiong M. Tao Y. Gao Q. Feng B. Yan W. Zhou Y.. (2021). Human stem cell-derived neurons repair circuits and restore neural function. Cell Stem Cell 28, 112–126.e6. doi: 10.1016/j.stem.2020.08.014, PMID: 32966778 PMC7796915

[ref177] Xu Z. Jiang H. Zhong P. Yan Z. Chen S. Feng J. (2016). Direct conversion of human fibroblasts to induced serotonergic neurons. Mol. Psychiatry 21, 62–70. doi: 10.1038/mp.2015.101, PMID: 26216300 PMC4518549

[ref178] Xu J. H. Qin X. Z. Zhang H. N. Ma Y. X. Qi S. B. Zhang H. C.. (2021). Deletion of Kruppel-like factor-4 promotes axonal regeneration in mammals. Neural Regen. Res. 16, 166–171. doi: 10.4103/1673-5374.286978, PMID: 32788472 PMC7818869

[ref179] Xu Z. Su S. Zhou S. Yang W. Deng X. Sun Y.. (2020). How to reprogram human fibroblasts to neurons. Cell Biosci. 10:116. doi: 10.1186/s13578-020-00476-2, PMID: 33062254 PMC7549215

[ref180] Yang Y. Chen R. Wu X. Zhao Y. Fan Y. Xiao Z.. (2019). Rapid and efficient conversion of human fibroblasts into functional neurons by small molecules. Stem Cell Rep. 13, 862–876. doi: 10.1016/j.stemcr.2019.09.007, PMID: 31631018 PMC6893066

[ref181] Yang L. Miao L. Liang F. Huang H. Teng X. Li S.. (2014). The mTORC1 effectors S6K1 and 4E-BP play different roles in CNS axon regeneration. Nat. Commun. 5:5416. doi: 10.1038/ncomms6416, PMID: 25382660 PMC4228696

[ref182] Yiu G. He Z. (2006). Glial inhibition of CNS axon regeneration. Nat. Rev. Neurosci. 7, 617–627. doi: 10.1038/nrn1956, PMID: 16858390 PMC2693386

[ref183] Yoo A. S. Sun A. X. Li L. Shcheglovitov A. Portmann T. Li Y.. (2011). MicroRNA-mediated conversion of human fibroblasts to neurons. Nature 476, 228–231. doi: 10.1038/nature10323, PMID: 21753754 PMC3348862

[ref184] Yoshida S. Kato T. M. Sato Y. Umekage M. Ichisaka T. Tsukahara M.. (2023). A clinical-grade HLA haplobank of human induced pluripotent stem cells matching approximately 40% of the Japanese population. Medicine 4:e10, 51–66.e10. doi: 10.1016/j.medj.2022.10.003, PMID: 36395757

[ref185] Yu J. Vodyanik M. A. Smuga-Otto K. Antosiewicz-Bourget J. Frane J. L. Tian S.. (2007). Induced pluripotent stem cell lines derived from human somatic cells. Science 318, 1917–1920. doi: 10.1126/science.1151526, PMID: 18029452

[ref186] Yue F. Cheng Y. Breschi A. Vierstra J. Wu W. Ryba T.. (2014). A comparative encyclopedia of DNA elements in the mouse genome. Nature 515, 355–364. doi: 10.1038/nature13992, PMID: 25409824 PMC4266106

[ref187] Zhang L. Yin J. C. Yeh H. Ma N. X. Lee G. Chen X. A.. (2015). Small molecules efficiently reprogram human Astroglial cells into functional neurons. Cell Stem Cell 17, 735–747. doi: 10.1016/j.stem.2015.09.012, PMID: 26481520 PMC4675726

[ref188] Zheng B. Tuszynski M. H. (2023). Regulation of axonal regeneration after mammalian spinal cord injury. Nat. Rev. Mol. Cell Biol. 24, 396–413. doi: 10.1038/s41580-022-00562-y, PMID: 36604586

[ref189] Zhu Y. Sousa A. M. M. Gao T. Skarica M. Li M. Santpere G.. (2018). Spatiotemporal transcriptomic divergence across human and macaque brain development. Science 362. doi: 10.1126/science.aat8077, PMID: 30545855 PMC6900982

[ref190] Ziffra R. S. Kim C. N. Ross J. M. Wilfert A. Turner T. N. Haeussler M.. (2021). Single-cell epigenomics reveals mechanisms of human cortical development. Nature 598, 205–213. doi: 10.1038/s41586-021-03209-8, PMID: 34616060 PMC8494642

